# Response of fluorescence morphs of the mesophotic coral *Euphyllia paradivisa* to ultra-violet radiation

**DOI:** 10.1038/s41598-019-41710-3

**Published:** 2019-03-27

**Authors:** Or Ben-Zvi, Gal Eyal, Yossi Loya

**Affiliations:** 1 School of Zoology, The George S. Wise Faculty of Life Sciences, Tel-Aviv University, Tel-Aviv, Israel; 2 The Interuniversity Institute for Marine Sciences in Eilat, Eilat, Israel; 3 ARC Centre of Excellence for Coral Reef Studies, The University of Queensland, Brisbane, Australia

## Abstract

*Euphyllia paradivisa* is a strictly mesophotic coral in the reefs of Eilat that displays a striking color polymorphism, attributed to fluorescent proteins (FPs). FPs, which are used as visual markers in biomedical research, have been suggested to serve as photoprotectors or as facilitators of photosynthesis in corals due to their ability to transform light. Solar radiation that penetrates the sea includes, among others, both vital photosynthetic active radiation (PAR) and ultra-violet radiation (UVR). Both types, at high intensities, are known to have negative effects on corals, ranging from cellular damage to changes in community structure. In the present study, fluorescence morphs of *E*. *paradivisa* were used to investigate UVR response in a mesophotic organism and to examine the phenomenon of fluorescence polymorphism. *E*. *paradivisa*, although able to survive in high-light environments, displayed several physiological and behavioral responses that indicated severe light and UVR stress. We suggest that high PAR and UVR are potential drivers behind the absence of this coral from shallow reefs. Moreover, we found no significant differences between the different fluorescence morphs’ responses and no evidence of either photoprotection or photosynthesis enhancement. We therefore suggest that FPs in mesophotic corals might have a different biological role than that previously hypothesized for shallow corals.

## Introduction

The solar radiation that reaches the earth’s surface includes, among others, ultra-violet radiation (UVR; 280–400 nm) and photosynthetically active radiation (PAR; 400–700 nm). As light travels through the atmosphere and penetrates the sea, it is scattered and reflected. At the end of this process, UVA, which ranges between 315 and 400 nm, is the portion of the UVR spectrum that demonstrates maximum penetration in seawater^1^. Owing to its short, high-energy wavelength, UVR is known to have mostly negative effects on living organisms. It is already established that UVR is one of the major stressors to coral reefs and, in combination with other stressors (i.e. elevated temperatures, bacterial infections, and pollution), it is responsible for coral bleaching and the degradation of coral reefs around the globe^2^. The effects of UVR or high intensities of PAR on corals and their symbionts are diverse: changes in community composition^3^, reduction in primary production^4,5^, breakdown of metabolic pathways, especially those of photosynthesis^6^, reduced growth^7,8^, and organismal mortality^9^. The indirect UVR-induced damage at the cellular or molecular level is caused by the formation of reactive oxygen radicals (ROS) that attack cellular compartments^10^. The direct UVR-induced DNA damage is caused by absorption of the high-energy wavelength by the DNA molecules that, in turn, can alter the structure of the DNA helix, leading to cell cycle arrest and cell death^11,12^. Two of the most abundant direct UVR-induced DNA lesions are those of cyclobutane pyrimidine dimers (e.g. CPDs) and 6–4 pyrimidine pyrimidone photoproduct (e.g. 6-4PPs)^13^. Such damage is highly significant, since it can occur in all organisms, from simple to complex. Being sessile organisms, corals have evolved the ability to accumulate UV-absorbing compounds, known as mycosporine-like amino acids (MAAs), which protect them from light-induced damages^14,15^. MAAs are known to efficiently absorb light in the UVR range^16^ and also to display moderate antioxidant activity^17^.

*Euphyllia paradivisa* is a large-polyp species usually found in shallow (<20 m), turbid lagoons^18,19^. In the Gulf of Eilat/Aqaba (GoE/A), however, its area of distribution is strictly mesophotic. Despite being found only at depths greater than 36 m in Eilat, this species has demonstrated both a low mortality rate and an ability also to prosper in shallow depths and shallow light environments for prolonged periods of time following transplantation^20^. When the photoacclimatization potential of this species to a shallow light environment was tested^21^, the corals lost their original mesophotic ability to efficiently utilize low-light intensities, but were still able to withstand higher light intensities before suffering from photoinhibition (Ben-Zvi *et al*.; in preparation). The high survivorship and successful photoacclimatization of *E*. *paradivisa* to high-light conditions tend to support the idea of mesophotic coral ecosystems (MCEs) serving as refugia or as a source of replenishment for the degraded shallow-coral ecosystems, a concept that was first presented by Glynn^22^ and postulated later as the “Deep Reef Refugia Hypothesis” (DRRH) by Bongaerts *et al*.^23^.

*E*. *paradivisa* also displays a dramatic color polymorphism as a result of its intense fluorescence. The fluorescence can be observed with the naked eye due to the mesophotic light environment, which naturally excites the fluorescent proteins (FPs). Corals can express one or multiple fluorescent and non-fluorescent chromoproteins^24–27^, and therefore alter their color morph during the course of different life stages^28^, under changing stressors or environmental conditions^29–31^, or along a depth gradient^32,33^. The plasticity of coral color phenotypes, which is partially a consequence of different types of FPs and the rapid regulation of the FPs^24^, may play a potential role in the biology of corals under changing environments. Two of the widely accepted and studied hypotheses regarding FP function in corals are: (1) a role in the enhancement of photosynthesis where light is scarce^34–36^; and (2) photoprotection under high-light conditions by means of altering the light inside the coral tissues^37,38^. Early studies on the photosynthesis enhancement hypothesis posited that FPs are able to absorb less suitable wavelengths for photosynthesis (<400 nm) and convert them to wavelengths that are more efficiently utilized by the zooxanthellae (>400 nm)^39,40^, or that they may provide light to shaded zooxanthellae by reflecting and scattering PAR^41^. A more recent study has presented evidence that a photoconvertible red FP (pcRFP) may provide longer wavelengths to the zooxanthellae present deeper in the coral host tissue^42^. Regarding their role in photoprotection, FPs have been shown to be upregulated by light^25^, specifically by blue light^24^, and to accumulate around the reproductive organs^43^ or light-exposed parts of the colony^44^. Furthermore, higher expression of red fluorescent protein (RFP) in *Acropora millepora* showed a correlation with reduced photo-damage in the algal symbionts^38^. We therefore sought to investigate here the possibility of UVR being the potential cause of *E*. *paradivisa*’s absence from the shallower reefs of Eilat, by monitoring the response of different fluorescence morphs under UVR stress. Additionally, we sought to test the two major hypotheses regarding FPs, in a mesophotic coral that displays fluorescence polymorphism.

## Results

### Fluorescence morphs

**Figure 1:**
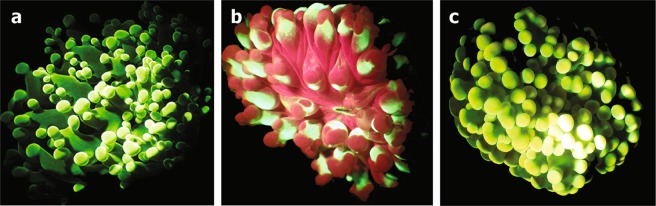
Three fluorescence morphs of the coral *Euphyllia paradivisa* from the Gulf of Eilat/Aqaba: (**a**) green (emission peaks at 480 nm and 505 nm), (**b**) red (emission peak at 505 nm), and (**c**) yellow (emission peaks at 505 nm and 545 nm).

*E*. *paradivisa* constituted up to 73% of the total coral cover at our sampling site (Dekel beach), Eilat^20^. Our field survey revealed that the most dominant fluorescence morph is the green morph. Out of 463 *E*. *paradivisa* polyps examined in the field survey, the green morph comprised 51.6% of the total number of polyps, the red morph 41.4%, and the yellow morph 5.4% (all other morphs together comprised 1.6% of the surveyed population). The spectral analysis of the excitation and emission peaks of the collected corals provided a better definition of the different morphs (Fig. 1). Emission peaks (for excitation at 450 nm) had been previously recorded for the species at 515 and 545 nm^26^. Here we report the green morph as presenting two fluorescence emission peaks, at 480 and 505 nm; the yellow morph also presented two peaks, at 505 and 545 nm; and the red morph presented one emission peak, at 505 nm. The red appearance of the latter morph originates from the fluorescence of chlorophyll (at 680 nm) and not from a RFP. The excitation peaks for the 480 nm, 505 nm, and 545 nm emitting FPs were 405 nm, 450 nm, and 520 nm, respectively.

### Response to UVR

**Figure 2:**
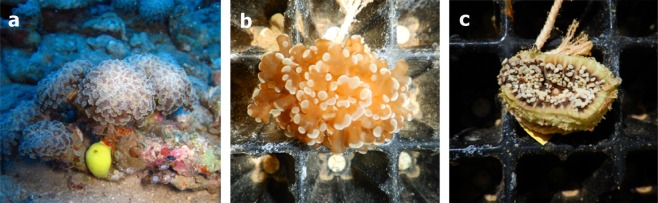
*Euphyllia paradivisa* tentacle extension (**a**) in their natural habitat at 45 m. (**b**) The same *E. paradivisa* polyp under PAR only, and (**c**) PAR + UV.

Both in nature and in aquaria, *E*. *paradivisa* has been observed to expand its tentacles during the day (Fig. 2a). Within a 24-hr period in the controlled light experiment, corals that were exposed only to PAR remained expanded during the light hours (Fig. 2b), while corals that were exposed to PAR + UV contracted fully into their skeleton (Fig. 2c). The contracted corals decreased in size by 73.1% ± 8.35 (mean ± SD, n = 6) compared to those exposed to PAR only, which even expanded slightly (7.94% ± 8.19, n = 6).

**Figure 3:**
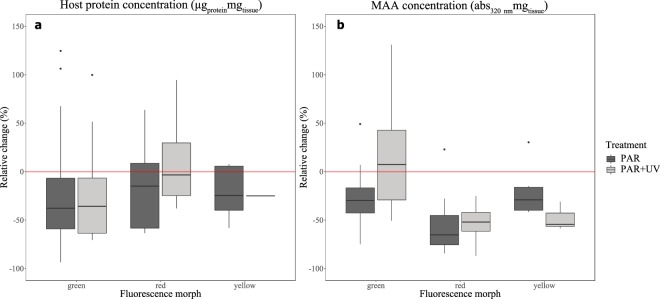
Changes in the physiology of *Euphyllia paradivisa* under two light treatments. The relative change (%) in (**a**) host protein and (**b**) MAA concentration of *E. paradivisa* under two light treatments: full sunlight (“PAR + UV”; light gray boxes) and PAR only (“PAR”; dark gray boxes), in three fluorescence morphs. Boxes represent the upper and lower quartile, center lines represent medians, and whiskers extend to data measurements that are less than 1.5*IQR away from first/third quartile. Outliers are represented by dots. Red lines represent a reference to the measurements taken prior to the light treatments.

Corals from all three morphs under both PAR and PAR + UV treatments presented a decrease in host protein concentration versus the measurement taken prior to the experiment, as indicated in Fig. 3a. There was no significant difference between treatments or morphs (LMM, F = 0.23, p = 0.632 and F = 1.22, p = 0.315, respectively; see material and methods for details). When examining the absorbance at 320 nm as a proxy for MAA concentration, we found that excluding the green morph under PAR + UV and a few outliers, the majority of the corals under both light treatments displayed a decrease in MAA concentration compared with measurements taken prior to the treatments. At the end of the experiment, corals under PAR + UV presented significantly higher mean ± SD absorbance at 320 nm (Fig. 3b; LMM, F = 4.164, p = 0.02) compared to corals under PAR only. When comparing the different morphs, the green and red morphs under PAR + UV treatment, presented higher MAA concentrations than under the PAR treatment while the yellow morph exhibited the opposite response (LMM, F = 9.641, p = 0.001).

**Figure 4:**
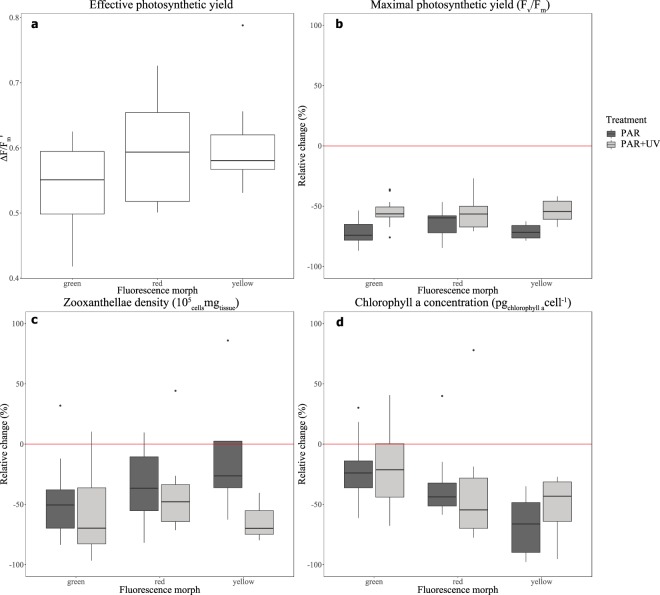
Photobiology of *Euphyllia paradivisa*. (**a**) Effective photosynthetic yield (${\rm{\Delta }}F/{{\rm{F}}^{\prime} }_{{\rm{m}}}$) of three fluorescence morphs of *E. paradivisa* in their natural habitat (45 m depth). Change (%) in the (**b**) maximal photosynthetic yield (F_v_/F_m_), (**c**) zooxanthellae density, and (**d**) chlorophyll a concentration under two light treatments: full sunlight (“PAR + UV”; light gray boxes) and PAR only (“PAR”; dark gray boxes), in three fluorescence morphs. Boxes represent the upper and lower quartile, center lines represent medians, and whiskers extend to data measurements that are less than 1.5*IQR away from first/third quartile. Outliers are represented by dots. Red lines represent a reference to the measurements taken prior to the light treatments.

The mean (±SD) effective photosynthetic yield (${\rm{\Delta }}F/{{\rm{F}}^{\prime} }_{{\rm{m}}}$) for *E*. *paradivisa* as measured in the field survey under ambient light at 45–50 m was 0.579 ± 0.073. We found no significant difference (one-way ANOVA, p = 0.094, n = 12 for each morph) between the photosynthetic yields of the different morphs in their natural habitat (Fig. 4a). In our controlled experiment, the maximal photosynthetic yields (F_v_/F_m_) of the corals dropped in both PAR and PAR + UV compared to the control measurement, from 0.696 ± 0.032 to 0.263 ± 0.09. F_v_/F_m_ values of polyps under the PAR + UV treatment were found to be ~1.5 fold higher than the PAR-treated polyps in all fluorescence color morphs (LMM, F = 18.306, p = 0.001), with a mean ± SD of 0.318 ± 0.069 under PAR + UV and 0.209 ± 0.076 under the PAR treatment (Fig. 4b). Zooxanthellae density presented with an opposite response in which the corals that were exposed to UVR had fewer zooxanthellae cells than corals who were deprived of UVR (Fig. 4c; LMM, F = 5.1, p = 0.027). Chlorophyll a concentration decreased under both light treatments but differed between treatments and morphs and showed no consistent response (Fig. 4d). In the latter two parameters (i.e. zooxanthellae density and chlorophyll a concentration) we observed a difference between morphs (LMM, F = 3.108, p = 0.071 and F = 2.899, p = 0.088, respectively). We noticed that the green morph showed the greatest decrease in zooxanthellae density (−53.14% ± 30.53) but also the smallest decrease in chlorophyll a concentration (−20.6% ± 26.15) and the yellow morph showed the smallest decrease in zooxanthellae density (−43.7 ± 99.93) and the highest decrease in chlorophyll a concentration (−61.41% ± 27.63).

### DNA damage

**Figure 5:**
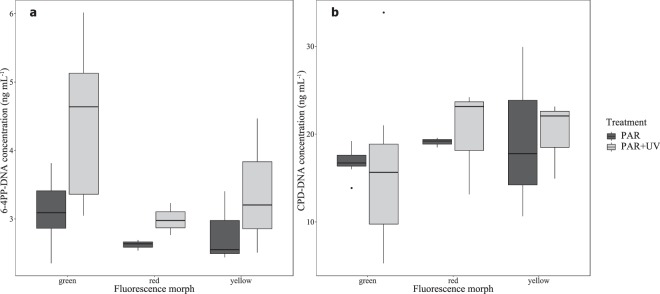
Quantification of UVR-induced DNA damage in *Euphyllia paradivisa*. (**a**) 6–4 photoproducts (6-4PPs) and (**b**) Cyclobutane pyrimidine dimers (CPDs) under two light treatments: full sunlight (“PAR + UV”; light gray boxes) and PAR only (“PAR”; dark gray boxes), in three fluorescence morphs. Boxes represent the upper and lower quartile, center lines represent medians, and whiskers extend to data measurements that are less than 1.5*IQR away from first/third quartile. Outliers are represented by dots.

Corals exposed to PAR + UV revealed a significantly higher accumulation of 6-4PP sites (Fig. 5a; LMM, F = 6.57, p = 0.016), and more, but not significantly, CPD sites than their UVR-deprived counterparts, specifically in the red and yellow morphs (Fig. 5b; LMM, F = 0.017, p = 0.898). There was no significant effect of the morph within each treatment on the amount of 6-4PP or CDP sites (LMM, F = 1.102, p = 0.346 and F = 0.086, p = 0.917, respectively).

## Discussion

In recent years there has been increasing interest in the DRRH^23^ and the potential of MCEs to serve as refugia for the detrimentally affected shallow corals or as a source for replenishment in case of shallow reef loss^45,46^. In addition, several recent studies have also focused on the uniqueness of the MCEs, rather than considering them as an extension of the shallow reefs^47,48^. In Eilat, *E*. *paradivisa* is a strictly mesophotic species and does not occur in the shallow reef, unlike other reefs in the world^18,19^. The absence of this species from the shallow reefs of Eilat might be explained by two possible scenarios: (1) its adaptation to the mesophotic, light-limited environment; and (2) local forces that drive it to the deeper part of the reef. Predation pressure is one suggested and tested explanation for the occurrence of this coral exclusively in the mesophotic reef of Eilat^20^. Here, we suggest that high-light intensities and UVR are other factors that exclude *E*. *paradivisa* from the shallow habitats in Eilat. In the present study, most of the measured physiological parameters (protein concentrations, zooxanthellae densities, and chlorophyll a concentration) dropped when the corals were exposed to higher light intensities than those found in their natural habitat in the GoE/A (both PAR and UVR intensities; Figs 3a and 4c,d). This implies that the mesophotic corals and their algal symbionts were suffering from acute light stress, presenting similar responses to those previously described for corals^10^. This result was not surprising in view of the known negative effects of high-light and UVR on corals. The photosynthetic apparatus is especially sensitive to reactive oxygen species (ROS) production following UVR exposure. Common recorded reactions to the production of ROS are damage to the photosynthetic membranes^49^, induction of photoinhibition^5,7^, and a decrease in photosynthetic efficiency^50^. Such typical outcomes following UVR exposure had been demonstrated in several shallow coral species that were either a-priori acclimated or not to high-light conditions^50^. High-light intensities or UVR exposure, usually along with elevated temperature, will also increase the tendency of corals to bleach and lose their zooxanthellae^51^. In this study, *E*. *paradivisa*, despite losing symbionts and chlorophyll a, displayed a higher maximal photosynthetic yield under the PAR + UV treatment compared to those that had been exposed to PAR only (Fig. 4b). However, corals from both treatments revealed a significantly lower photosynthetic yield compared to the values at the beginning of the experiment, and to that measured in their natural habitat (Fig. 4a,b). One potential explanation for the less impaired photosynthetic efficiency under UVR is that of the coral’s distinct behavioral reaction observed during the experiment (Fig. 2). *E*. *paradivisa* is a fleshy tissue coral, somewhat sea anemone-like, and able to contract almost completely into the skeleton. Corals under UVR exposure almost fully contracted throughout the duration of the experiment (Fig. 2c). This behavior was also observed by Siebeck^52^ in several massive shallow-coral species but has not been previously described in mesophotic corals. We suggest that this reaction might be triggered by a UVR receptor that has either been found or suggested in other organisms^53,54^. Such a receptor might have enabled the protection of the remaining zooxanthellae from the intense radiation, and consequently mitigated their impairment. Although this might be helpful as a short-term protection mechanism against UVR damage, it nonetheless did not prevent the loss of zooxanthellae and chlorophyll and the occurrence of DNA damage, nor did it prevent the corals from containing more MAAs under UVR exposure. Hence, we assume that the corals would have not survived for a prolonged light stress experiment while contracted.

Another studied mechanism of UVR damage avoidance is that of MAAs accumulation. Corals and other marine organisms have been shown to accumulate UV-absorbing MAAs when exposed to UVR^14,55^, thereby improving their ability to mediate excess light. Although presenting a decrease in MAA concentration (Fig. 3b) compared to the measurements taken prior to the experiment (excluding the response of the green morph), the corals indeed presented higher MAA levels when exposed to UVR, despite the short-term exposure of eight days and the fact that these corals are exposed to only minor levels of UVR at the mesophotic depths^20^. The differences in MAA concentration found among the fluorescent morphs might also be accompanied by a change in the composition of MAAs among these morphs, as demonstrated in *Porites astreoides*^8^. Corals have a rapid and efficient DNA repair mechanism compared to other organisms^56^, but since they were sampled during midday, we infer that the results represent the maximal damage of an average day. Both CPDs and 6-4PPs result in the deformation of the DNA helix, which causes a downstream effect. Our results demonstrate that the corals that were exposed to UVR suffered from approximately two-fold more 6-4PP sites and that there was no difference in the DNA damage between the morphs (Fig. 5). While known to be able both to photoacclimatize to shallow habitats^20^ and to be found in those habitats (5–20 m)^18,19^, here we demonstrated that *E*. *paradivisa* suffered from light stress, especially in the presence of UVR. The previous successful transplantation of this coral to shallow environments was performed on adult corals that were kept covered by a mesh or by lighting filters, assumedly reducing the light received by them^20^. Furthermore, the UVR or high-light intensities found in the shallow reefs of Eilat may not be fatal for adult corals but might be fatal for *E*. *paradivisa* recruits or juveniles. Therefore, UVR or high-light should be considered as a factor that drives *E*. *paradivisa* and other species to the deeper mesophotic reefs of Eilat.

Previous studies have shown that different non-fluorescent color morphs are correlated to differences in stress response, such as different compositions of MAAs^8^, changes in photodamage^38^, and differences in photosynthetic yield^57^. Considering the FP ability to manipulate light, we therefore sought to explore the physiological response of different fluorescent morphs in corals. In most of our measurements, we found no difference between the different fluorescence morphs under either light treatment. Moreover, we assume that *E*. *paradivisa* is not subjected to any high-light or UVR stress in its native habitat, and none of the excitation peaks found here is in the range of UVR. Consequently, the reason for the intense fluorescence and fluorescence polymorphism displayed by *E*. *paradivisa* may not be related to photoprotection, unlike that suggested for shallow corals^37,38^. The relationship between FPs and photosynthesis at the mesophotic depth has also been previously investigated, but measurements were not performed under ambient mesophotic light environment^32^. This study, in contrast, presents *in-situ* measurements of photosynthetic yield under the unique and well-defined light environment of the mesophotic reef in Eilat^20^. As no differences were found in the photosynthetic performances among the morphs in the field survey or in the controlled experiment, there is also no evidence of photosynthesis enhancement by the coral FPs, as also suggested for other shallow and mesophotic corals^32,58^. The lack of evidence supporting this role in mesophotic environments was previously demonstrated in *Leptoseris* spp. by Roth *et al*.^32^. In the case of *E*. *paradivisa*, the role of fluorescence remains unknown and other hypotheses, such as prey attraction^59^, inflammation-like response^60,61^, and antioxidant activity^62^, should be further tested.

## Materials and Methods

### Field surveys and measurements

Belt transects (50 m × 0.5 m) were surveyed using closed-circuit rebreather dives at the collection site at Dekel Beach, GoE/A at 45 m (29°32′20.02″N 34°56′44.80″E), where *E*. *paradivisa* is most abundant^20^. Transects were photographed and analyzed as follows: each polyp within each transect was categorized as one of five morphs (green, yellow, red, red-green, and orange). The abundance (%) of each morph was calculated by dividing the total number of polyps of each morph by the total number of polyps (463) in the transect. Effective photosynthetic yield (${\rm{\Delta }}F/{{\rm{F}}^{\prime} }_{{\rm{m}}}$) of 12 colonies from three different fluorescence morphs (n = 12 for each morph; green, red, and yellow) was measured at the survey site using a Diving-PAM (Walz, Germany). Measured colonies were at least 1.5 m away from each other, therefor the colonies were genetically distinct, with high certainty, from one another. Corals were measured during the day under ambient light, and “measuring light intensity” and “gain” values were adjusted to reach optimal signals followed by an auto-zero calibration in site.

### Coral sampling and experimental design

Fourteen colonies (ca. six polyps in each colony) from three fluorescent morphs of the coral *E*. *paradivisa* (i.e. “green” n = 8, “yellow” n = 3, and “red” n = 3 colonies) were sampled under permit 015/41127 of the Israel Nature and Parks Authority, from Dekel Beach and transferred to running seawater aquaria under a filter that mimics light conditions of ca. 40 m (“Lagoon blue”, Lee Filters) at the Interuniversity Institute in Eilat (IUI). Each colony was fragmented into individual polyps, resulting in 49 green polyps, 13 yellow polyps, and 17 red polyps. Tentacles from each polyp were sampled prior to exposure to the light treatments, weighted, and preserved at −80 °C for physiology analyses, following a 5 sec blotting on an absorbing tissue, and flash-freezing in liquid nitrogen. The polyps were then assigned, with equal representation from each colony, to one of two light treatments: (1) full ambient sunlight (i.e. PAR + UV treatment), receiving 1,624 ± 69 µmol m^−2^ s^−1^ of PAR and 117 ± 16 µmol m^−2^ s^−1^ of UVA and UVB (mean ± SD at 1200 hours during the experiment); or (2) full ambient sunlight but covered with a UV absorption filter (Ultra-Violet Absorption, Lee Filters) that cuts the light spectrum below 400 nm (see Supplementary Fig. SI1) therefore receiving only the PAR portion of the spectrum (i.e. PAR treatment). Each treatment contained three independent tanks with individual water inlet. Mean (±SD) seawater temperature during the experiment was 21.6 ± 0.22 °C as measured by a data logger (HOBO, Onset computer corporation). Corals were exposed to the different light treatments for eight consecutive days and tentacles were sampled again at midday (1200 hours) on Day 8 and were preserved for DNA damage analysis in RNA Save stabilization solution (Biological industries, Israel) and for physiology.

### Behavioral experiment

Following Day 8 of the experiment, and in order to quantify an observed contraction of the tentacles, a second experiment was set up with six new polyps (two from each fluorescence morph). The polyps were subjected first to PAR treatment for 24 hr, followed by 24 hr under the PAR + UV. After 48 hr of recovery under the Lagoon blue filter, the polyps were first exposed to PAR + UV for 24 hr followed by 24 hr under PAR only. The polyps were photographed at the beginning and end of each exposure, the top-projection area of tentacles was measured using Photoshop software, and contraction/extension percentage was calculated by dividing the area at the end of the exposure by the area at the beginning of the exposure.

### Spectral analysis of fluorescence color morphs

Fluorescence excitation and emission peaks of the three fluorescence color morphs used for the experiment were determined from the collected colonies using the Varian Cary Eclipse Fluorescence Spectrometer (Agilent, USA) from host protein extracts.

### Coral physiology

Coral tissue was thawed on ice and homogenized mechanically. Host tissue and zooxanthellae were separated by a second homogenization and centrifuging the tissue at low speed (5,000 rpm for 5 min). The host supernatant was centrifuged again at high speed (14,000 rpm for 5 min) to cleanse the supernatant of any additional zooxanthellae prior to the host protein concentration analysis. The symbiont pellet was used for zooxanthellae density calculations and chlorophyll concentration analysis. Since *E*. *paradivisa* is a fleshy coral with high tissue volume, and only coral tentacles were sampled, the common methods for determining coral surface area (e.g., the paraffin or aluminum foil methods) are irrelevant in this case. Protein and MAA concentrations were therefore normalized to tissue net wet weight of the sampled tentacles following removal of the excess water in the sample. Chlorophyll a concentrations were normalized to zooxanthellae cells.

#### Host protein concentration

Host protein concentrations were measured in triplicate using the Coommassie blue assay (Thermo Fisher Scientific, USA) according to the manufacturer’s protocol. The standard microplate protocol (100–1500 µg/ml) was used based on previous experience with *E*. *paradivisa*, and absorbance was read with a Multiskan microplate spectrophotometer (Thermo Fisher Scientific, USA).

#### Mycosporine-like Amino Acid (MAA) concentration

Absorbance at 320 nm of the host protein extractions was measured as approximation for MAA relative concentration, using a Multiskan microplate spectrophotometer (Thermo Fisher Scientific, USA). Since the maximum absorbance of MAAs in *E*. *paradivisa* had not been examined yet, absorbance at 320 nm was used as described by Shibata^63^.

### Photobiology

Zooxanthellae cells were counted in triplicate under a light microscope using a hemocytometer in a 1:10 dilution. Chlorophyll was extracted using 90% acetone for 15 hr incubation at 4 °C and concentrations were determined using a Multiskan microplate spectrophotometer (Thermo Fisher Scientific, USA) and the equations previously described by Jeffery and Humphrey^64^. Maximal photosynthetic yield (F_v_/F_m_) was measured using a Diving-PAM (Walz, Germany) for each polyp. Polyps were measured approximately 90 min after sunset to ensure a proper dark acclimation state prior to the measurement, both prior to their exposure to the light treatments and at the end of the experiment. The “measuring light intensity” and “gain” values were adjusted to reach optimal signals followed by an auto-zero calibration.

### UV-induced DNA damage

Total DNA was extracted using DNeasy Blood & Tissue kit (Qiagen, Germany) according to the manufacturer’s protocol. DNA concentrations were assessed spectrometrically and pools of DNA were created from polyps of the same colony under the same treatment (three polyps in each pool). UV-induced DNA damage was quantified using OxiSelect™ UV-Induced DNA Damage ELISA Kits (Cell Biolabs, Inc., USA) according to the manufacturer’s protocol. This method of DNA damage quantification is based on specific primary antibodies that target either 6-4PPs or CPD sites, and secondary antibodies that create a color reaction that can be quantified spectrometrically.

### Statistical analyses

All statistical analyses were conducted with the statistical computing language R, version 3.5.1^65^. *In-situ* effective photosynthetic yield data were tested with one-way ANOVA, examining the effect of morph on the ∆F/Fm′. In all other analyses (except DNA damage, where we did not have a measurement prior to the light treatments), the change between the measurements taken both prior to and after exposure to the different light treatments was calculated and considered as the dependent variable. In order to account for the effect of both treatment and morph, while also considering the possible effect of the polyp genotype (colony) and the multiple tanks in each of the treatments, data were tested with linear mixed effects model (LMM) using lme4 package version 1.1^66^ and lmerTest^67^. Treatment (PAR vs. PAR + UV) and morph (green, red, and yellow) were treated as crossed fixed effects while colony (nested within morph) and tank (nested within treatment) were considered as random effects. LMM analyses were followed by ANOVA tests between each full model and a model lacking either treatment or morph. Differences were considered significant for a p-value < 0.05. Normality and homogeneity of variance of the models’ residuals were inspected visually.

## Supplementary information

Supplementary Information

## Data Availability

The datasets generated during and/or analyzed during the current study are available in the GitHub repository, https://github.com/orbzvi/Response-of-fluorescence-morphs-of-the-mesophotic-coral-Euphyllia-paradivisa-to-ultra-violet-radiati.
