# Supplementary material for: Response of fluorescence morphs of the mesophotic coral Euphyllia paradivisa to ultra-violet radiation
Source: Sci Rep. 2019 Mar 27;9:5245. doi: 10.1038/s41598-019-41710-3 (PMC6437176; doi:10.1038/s41598-019-41710-3)
Supplement: Supplementary file 1 — Supplementary Information [file 41598_2019_41710_MOESM1_ESM.pdf]

Supplementary Information

# Response of fluorescence morphs of the mesophotic coral *Euphyllia paradivisa* to ultra-violet radiation

Or Ben-Zvi[1,2], Gal Eyal[3], Yossi Loya[1]

[1] *School of Zoology, Faculty of Life Sciences, Tel-Aviv University, Israel*

[2] *The Interuniversity Institute for Marine Sciences of Eilat, Israel*

[3] *ARC Centre of Excellence for Coral Reef Studies, The University of Queensland, Australia*

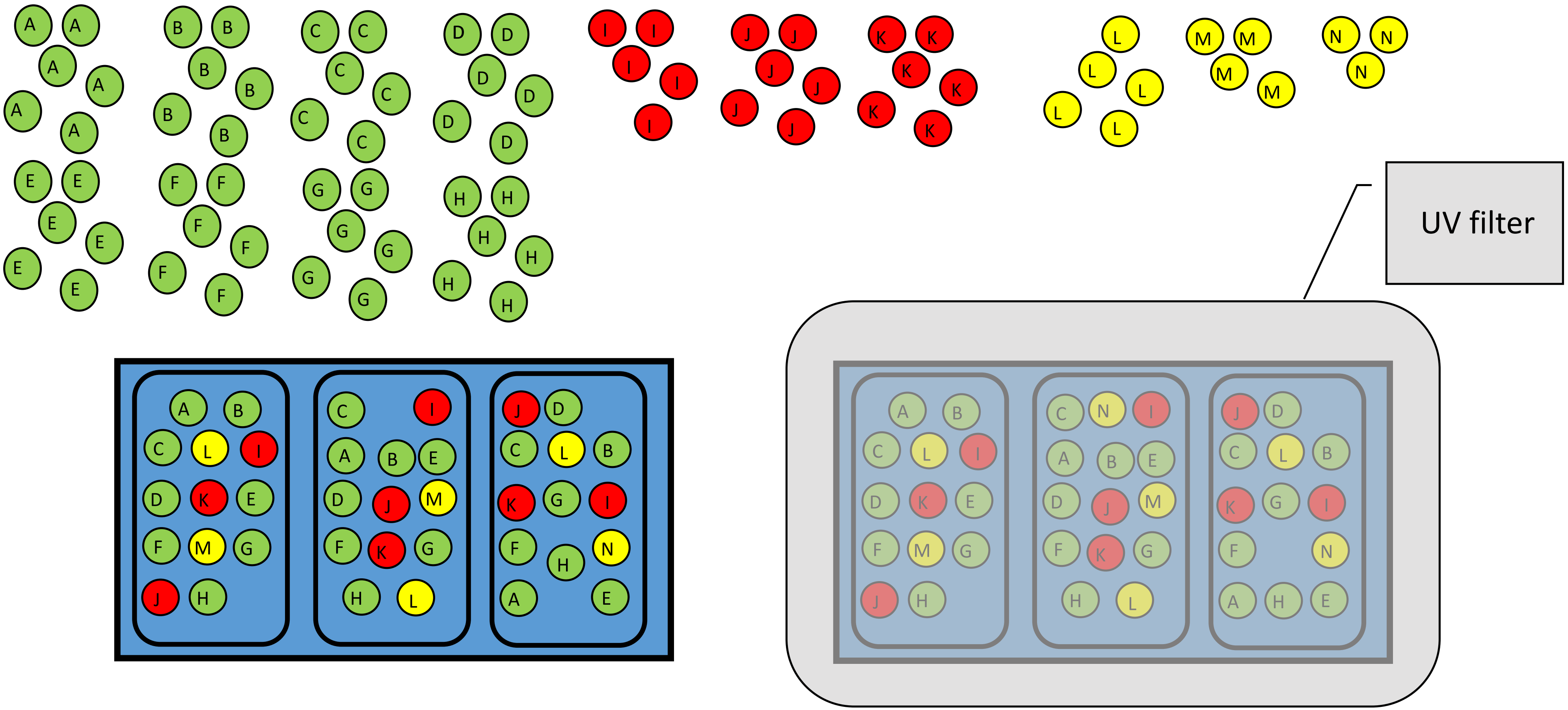


SI 1. Experimental design. Fourteen colonies (~six polyps in each colony) from three fluorescence morphs of the coral *Euphyllia paradivisa* (i.e. “green” n=8, “yellow” n=3, and “red” n=3 colonies from each morph) were sampled from the Dekel beach and transferred to running seawater aquaria under a filter that mimics light conditions at ~40 m (“Lagoon blue”, LEE Filters) at the Interuniversity Institute for Marine Sciences in Eilat (IUI). Each colony was fragmented into individual polyps resulting in: n=49 green polyps, n=13 yellow polyps, and n=17 red polyps. The polyps were then assigned, with equal representatives from each colony, to one of two light treatments; (1) full ambient sunlight (i.e. PAR+UV treatment); or (2) full ambient sunlight but covered with a UV absorption filter (“LEE U.V.”, LEE Filters) that cuts the light spectrum below 400 nm.
